# 2022 Malaysian Working Group Consensus Statement on Renal Denervation for management of arterial hypertension

**DOI:** 10.1038/s41440-022-00937-w

**Published:** 2022-06-01

**Authors:** Yook Chin Chia, Wan Azman Wan Ahmad, Alan Yean Yip Fong, Azhari Rosman, Abdul Rashid Abdul Rahman, Gim Hooi Choo, Soo Kun Lim, Mohammad Zawawi Abu Bakar, Tiong Kiam Ong

**Affiliations:** 1 Department of Medical Sciences, School of Medical and Life Sciences, Sunway University, Bandar Sunway, Selangor Malaysia; 2 Department of Primary Care Medicine, Faculty of Medicine, University of Malaya, Kuala Lumpur, Malaysia; 3 Department of Medicine, University Malaya Medical Centre, Kuala Lumpur, Malaysia; 4 Department of Cardiology, Sarawak Heart Centre, Kota Samarahan, Sarawak General Hospital, Kuching, Malaysia; 5 Clinical Research Centre, Institute for Clinical Research, Sarawak General Hospital, Kuching, Malaysia; 6 National Heart Centre, Jalan Tun Razak, Kuala Lumpur, Malaysia; 7 An Nur Specialist Hospital Bandar Baru Bangi, Bangi, Selangor Malaysia; 8 Cardiac Vascular Sentral, Jalan Sentral, Kuala Lumpur, Malaysia; 9 Department of Medicine, Faculty of Medicine, University of Malaya, Kuala Lumpur, Malaysia; 10 Klinik Kesihatan Gunung Rapat, Ipoh, Perak Malaysia

**Keywords:** blood pressure, consensus, uncontrolled resistant hypertension, Malaysia, renal denervation

## Abstract

Hypertension is highly prevalent and a major contributor to cardiovascular mortality and morbidity. In spite of the availability of efficacious, safe and affordable anti-hypertensive drugs, hypertension remains poorly controlled in the majority of hypertensive patients. Various reasons including non-adherence to the anti-hypertensive drugs, account for the poor control. Resistant hypertension is also one of the reasons for poor control of blood pressure (BP). The sympathetic nervous system (SNS) has long been recognized as one of the determinants in the pathophysiology of a raised BP. Overactivity of the SNS is a contributor to sustained arterial hypertension. Renal denervation (RDN) is increasingly recognized as a safe and effective adjunctive therapy to control BP with or without pharmacotherapy. Hence for patients who remain uncontrolled despite all efforts, renal denervation (RDN) is a novel treatment that can potentially improve BP control, hence reducing the major adverse cardiovascular events (MACE). More recent randomized, sham control trials of RDN have shown that RDN produces a sustained lowering of BP. To date, this lowering of BP through RDN is maintained for at least 3 years. Furthermore, this procedure has been found to be safe. Hence this consensus summarises the science behind RDN and the available clinical data to support the use of this therapy. It is hoped that this consensus will offer guidance on the importance of identifying patients who will benefit most from this therapy. A multidisciplinary team approach in the management of the patient undergoing RDN is recommended.

## Graphical abstract

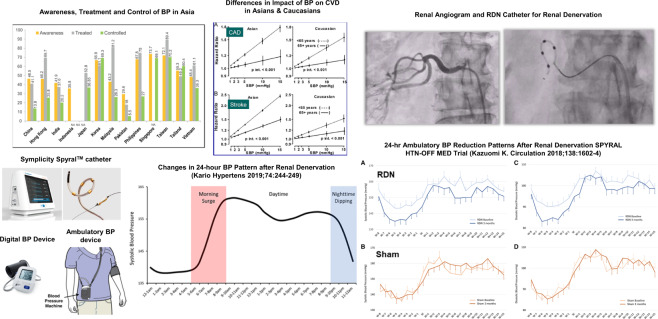

## Introduction

The latest Malaysian National Health and Morbidity Survey 2019 showed that 30% of Malaysians above the age of 18 years suffer from hypertension [1]. Numerous studies in different population groups demonstrated that optimal blood pressure (BP) control was achievable in only about a third of hypertensive patients. There are many reasons for this, including under-diagnosis, clinician inertia and disease progression. But the most important reason is probably patient non-adherence because the disease is asymptomatic in the early stages and treatment is lifelong.

Despite their limitations, lifestyle modification combined with pharmacotherapy are the mainstay of hypertension management. Patient counselling should be aggressively pursued to ensure or improve adherence and control of BP.

More than half of Malaysian hypertensive patients have a BP that remained uncontrolled despite the best possible pharmacological intervention [1]. For these patients, renal denervation (RDN) is a novel treatment that can potentially improve their BP control in a way that is not wholly dependent on long-term treatment adherence.

Various guidelines define slightly different BP targets [2–4]. This will of course influence the reported rates of uncontrolled hypertension - being higher when the BP goals are more stringent. The Malaysian Clinical Practice Guidelines on Hypertension and the latest WHO guideline defined a BP goal of <140/90 mmHg. [4, 5].

Reasons for uncontrolled hypertension abound—from patient, physician-related as well as system-related factors.

There is also a subset of patients in whom BP remains uncontrolled despite earnest adherence to guideline directed therapies. The categorization as resistant hypertension requires a failure to achieve BP goal despite treatment with at least 3 medications (inclusive of a diuretic). All 3 drugs should have been given at maximally tolerated doses and the patients had been adherent to medications, as well as pseudo-resistance and secondary causes of hypertension have been excluded [2–4, 6].

The prevalence of resistant hypertension has been consistently reported as 10–20% of all persons with hypertension [7, 8]. In Malaysia, a cross-sectional survey in a single centre outpatient department found 8.8% out of 1217 patients to have resistant hypertension [9]. Another survey amongst elderly hypertensives in 2 primary care clinics in the Klang Valley noted, not unexpectedly a higher prevalence rate of 66.3% of resistant hypertension as this study included only hypertensive patients who were elderly and aged 65 years or older while the other study above included younger adults from age 18 years and above. Furthermore, this study involving only elderly patients, reported that in their multivariate analysis to identify the determinants of resistant hypertension, older age was an independent determinant of resistant hypertension [10].

The sympathetic nervous system of the kidneys plays an important role in the pathophysiology of hypertension. In patients with hypertension, chronic activation of the efferent and afferent pathways produces pathological changes such as increased renin secretion, salt and water retention, peripheral vasoconstriction, left ventricular hypertrophy and cardiac arrhythmias. RDN can potentially reverse some of the pathological changes associated with hypertension [11] and may have particular and important clinical implications given the Asian characteristics of a higher salt intake, higher salt sensitivity, higher morning surge and the differences of the impact of BP on CV disease in Asians compared to the Caucasians [12, 13].

The efficacy and safety of RDN have been demonstrated in many clinical trials. Overall, current clinical evidence especially from recently concluded randomized controlled trials show that RDN does work in selected hypertensive individuals. The quantum of BP lowering is at least equivalent to that achievable with a single anti-hypertensive medication. In fact, the Global SYMPLICITY Registry (GSR), the sub-study of the GSR in South Korea and several other studies with longer durations of follow-up suggested that there is a potential for further BP reductions over time [14–18].

Point of view

- Clinical relevance
  Positioning the role of RDN in Asian hypertensive patients
- Future direction
  To determine the role of RDN in the management of young moderate hypertensive patients in addition to pharmacotherapy in Asia
- Consideration of RDN for the Asian population
  - Young hypertensives with overactive sympathetic drive as main mechanism for high BP
  - Patients with comorbidities
  - Patients with target organ damage
  - Patients on polypharmacy for multiple comorbidities
  - Patients who are non-adherent/non-compliant

## Patient preference

Medication adherence is low amongst younger hypertensives, those on polypharmacy especially without the use of fixed-dose combinations and patients who develop medication-related side effects [19, 20].

Patients’ regard for RDN often differs from their physicians’ perspective. This preference for RDN need not necessarily happen only for those with high pill burden or very severe hypertension [21–23].

The preference for RDN to improve blood pressure control without having to resort to medications needs to be discussed with considerations of anticipated benefits, safety, cost, and procedural acceptance.

## Factors that may influence diagnosis of resistant hypertension

Pseudo-resistant hypertension is a term used to describe a situation where uncontrolled BP is not truly resistant to anti-hypertensive medications but results from factors that may influence BP readings independent of pharmacological treatment of hypertension [24].

A significant proportion of uncontrolled resistant hypertension is due to factors that can cause a falsely elevated BP in patients already taking three or more types of anti-hypertensive medications. These include improper BP measurement techniques, medication non-adherence, white coat hypertension, undertreatment and clinical inertia [6, 20, 25–29].

A poor BP measurement technique is common in a busy clinical setting. Common mistakes include a single measurement of BP, an inappropriate BP cuff size, placement of BP cuff over thick clothing, patient not adequately rested, talking during BP measurement and incorrect body position.

A prospective study in a hypertension specialty clinic, patients with apparent resistant hypertension who were referred for further management were analysed. BP measurements taken during triage were compared with BP measurements taken by a trained physician under more controlled situations and with appropriate BP measurement techniques. The systolic and diastolic BP readings were 33/21 mmHg higher during triage compared with standardized measurement with a median difference of 23/13 mmHg. This study demonstrated that 33% of patients who were referred would have been misdiagnosed as having resistant hypertension based solely on their initial triage assessment [6].

Poor adherence to anti-hypertensive medications is one of the major causes of uncontrolled BP and is a common phenomenon in patients with chronic diseases. The World Health Organization had reported that the medication adherence rate in hypertensive patients was only 50% in developed countries and even lower in developing countries due to poor accessibility to medication and health care services [20, 29]. A local study on medication adherence in hypertensive patients showed similar results. In this retrospective study of hypertensive patients in a government primary care clinic setting, only 53% of patients were adherent to treatment. High total number of drugs and multiple daily doses had negative impacts on adherence [30].

White coat hypertension is another important cause of pseudo-resistant hypertension. In this condition, office or clinic BP is uncontrolled but out-of-office BP measurement shows controlled BP in patients on ≥ 3 anti-hypertensive agents. This condition is illustrated in a study where 8295 cases of apparent resistant hypertension were included in an ambulatory BP monitoring registry. The study demonstrated that only 62.5% of patients had sustained uncontrolled hypertension both during clinic visit and during ambulatory BP monitoring. The other 37.5% had white coat hypertension [8]. This was consistent with the findings of a study done in Malaysia which included patients with resistant hypertension referred for renal denervation therapy. The study showed 35% of patients had white coat hypertension confirmed with ambulatory BP monitoring [31]. Thus, the white coat effect is common in patients with apparent resistant or uncontrolled hypertension as seen in several studies based on home blood pressure measurements [25, 26]. Further evaluation with ambulatory BP monitoring or validated automated home BP devices is vital for an accurate diagnosis of true resistant hypertension.

Clinical inertia or failure of health care providers to initiate or intensify the treatment regime when indicated may lead to suboptimal control of BP [27, 28]. In a large cohort from more than 200 community-based outpatient clinics in the United States, 147,635 or 31.5% were diagnosed as having resistant hypertension. However, only 15% of these patients received optimal anti-hypertensive medications [32]. A study in a public primary care clinic in Penang, Malaysia showed a 19.2% prevalence of clinical inertia in the uncontrolled BP group. In fact, clinical inertia increased the odds of uncontrolled hypertension by 7.82 times [33].

A comprehensive review of patient’s medications including over the counter drugs, supplements and herbs is important in the assessment of uncontrolled resistant hypertension. Medications that are known to cause BP elevation include NSAIDS, combined hormonal contraceptives, corticosteroids, antineoplastic agents, anti-depressant (venlafaxine hydrochloride), liquorice, immunosuppressive agents (cyclosporine, tacrolimus), erythropoietin, antiretroviral treatment, alcohol, caffeine, cocaine and salt-containing medications [34].

**Fig. 1:**
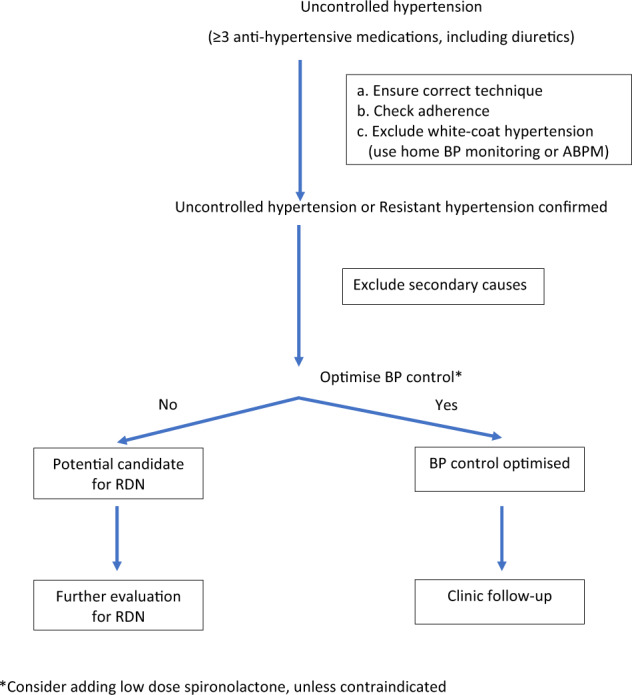
How do we identify patients who need RDN—a flow chart. *Consider adding low dose spironolactone, unless contraindicated

It is important to identify and select the appropriate patients who will benefit most from RDN. RDN can be considered for particular patients or patient situations (Table 1 and Fig. 1) Particular emphasis is made with regard to patient selection as it is a key determinant of RDN suitability and success.

**Table 1:** Potential patients for RDN

1. BP remains high or above target despite full adherence with the maximum appropriate combination of pharmacological agents that can be tolerated.
2. Resistant hypertension, i.e., failure to achieve office BP goal (<140/90 mmHg) despite treatment with at least 3 anti-hypertensive medications (inclusive of a diuretic).
3. History of repeated non-adherence despite numerous counselling sessions about the risks and long-term consequences of poorly controlled hypertension.
4. On polypharmacy for multiple comorbidities and consequently facing risks of taking the wrong drug, the wrong dose, and drug-to-drug interactions.
5. Multiple end-organ damage, with high cardiovascular risk.
6. Unwilling to take long-term pharmacotherapy
7. Intolerance to anti-hypertensive medications.
8. Secondary causes of hypertension have been treated but BP remains uncontrolled.
9. Hypertension believed to be due to a hyperactive renal sympathetic system, e.g., nocturnal hypertension, early morning hypertension, hypertension associated with a relative resting tachycardia and obstructive sleep apnoea.
10. Repeated admissions for hypertensive crises.

### Clinical evidence

Renal denervation (RDN), initially with radiofrequency ablation, as a treatment for hypertension was initially explored in the 1990s, and early clinical data was published just over a decade ago, followed by many other studies and reviews done more recently [35–42]. Initial promise led to methods other than radiofrequency ablation, namely ultrasound and neurotoxic substances, as well as trials evaluating different indications for RDN being developed [42–45]. More work has been done more recently to provide the evidence of the efficacy and safety of RDN, particularly in well-designed sham control randomised control trials [40, 41, 44–46]. However, despite good safety signals, there was no single technique then that showed marked superiority over one another. The first sham-controlled trial for renal denervation commenced in 2014—the SYMPLICITY HTN-3, and the results did not show a statistically significant difference in BP reduction between the RDN and sham procedure [47]. While there were many factors that could have affected this, a lot of RDN research activities were, at that time, temporarily suspended or permanently terminated. Amongst factors identified were a large heterogenous study population, issues pertaining to changes in prescribed medications and also technical issues, whereby less than 10% of procedures were performed to the recommended technique[48]. With this knowledge, improved technology and trials were developed. The SPYRAL OFF-MED, SPYRAL ON-MED and RADIANCE-HTN SOLO were at the vanguard of these new trials [49, 50].

The SPYRAL OFF-MED proof-of-concept study was a single-blind (participant) multicentre randomised, sham-controlled study, of 80 patients with mild to moderate hypertension, off anti-hypertensive medications, using radiofrequency ablation delivered by the SYMPLICITY SPYRAL catheter. The primary endpoint was a change in ambulatory (systolic) BP measurement (ABPM) after 3 months. A statistically significant 5 mmHg drop in 24-hour ABPM and a 7.7 mmHg drop in office systolic BP, in favour of RDN was seen [44]. Subsequently, the SPYRAL OFF-MED Pivotal trial with a larger number of patients enroled (renal denervation [n = 166] or a sham procedure [n = 165]) showed a treatment difference between the two groups for 24-h systolic blood pressure of −3·9 mmHg and for office systolic blood pressure of −6·5 mmHg in favour of RDN [51].

The RADIANCE-HTN SOLO was also a single-blind (participant) multicentre randomised, sham-controlled study, of 146 patients with mild to moderate hypertension, off anti-hypertensive medications, using ultrasound emissions from the PARADISE balloon cooled ultrasound catheter. The primary endpoint was a change in systolic daytime 24-h ABPM after 2 months. Again, a statistically significant 6.3 mmHg drop of daytime ABPM and 6.5 mmHg drop in office systolic BP, in favour of RDN was seen [52].

The SPYRAL ON-MED proof-of-concept trial was, again, a single-blind (participant) multicentre randomised, sham-controlled study, of 80 patients with mild to moderate hypertension, on 1–3 anti-hypertensive medications, using radiofrequency ablation delivered by the SYMPLICITY SPYRAL catheter. The primary endpoint was a change in 24-h ABPM after 6 months. A statistically significant 7.4 mmHg drop in 24-hour ABPM and a 6.8 mmHg drop in office systolic BP, in favour of RDN was seen [45]. Since then the long-term efficacy and safety of this proof-of-concept study results reported that the initial BP reductions seen at 6 months was even greater and sustained as at 36 months; the ambulatory systolic blood pressure reduction was—18·7 mmHg (SD 12·4) for the renal denervation group (*n* = 30) and –8·6 mmHg (14·6) for the sham control group (*n* = 32); adjusted treatment difference was –10·0 mmHg, 95% CI –16·6 to –3·3; (*p* = 0·0039) and there were no short-term or long-term safety issues associated with renal denervation [16]

Another study of similar design, using a VESSIX catheter, the REDUCE-HTN: REINFORCE, in a similar patient group of mild to moderate hypertension, was inconclusive [53]. Reassuringly, this and the recently completed and published trials above demonstrated the safety profile of RDN.

More recently, the RADIANCE-HTN TRIO, a single-blind (participant) multicentre randomised sham-controlled study, of 136 patients with hypertension resistant to a standardised triple pill combination, demonstrated that using the PARADISE balloon cooled ultrasound catheter could significantly reduce daytime ambulatory systolic BP by 4.5 mmHg [54].

Finally, the magnitude of BP reductions, including that demonstrated by RDN therapy, at both ABPM and office systolic blood pressure readings, translates to improved cardiovascular outcomes [55, 56]. The clinical relevance of these results i.e. a decrease of nearly 10 mmHg in office systolic BP reading is estimated to lead to relative risk reductions of 17% for cardiovascular disease, 28% for heart failure, 20% in the cardiovascular event rate, and 13% in total mortality [57, 58]. In Malaysia, such effect would further translate to reductions in hospital admissions and bed-occupancy days, particularly in the heavily subsidized public health care sector [59].

The theoretical considerations to offer RDN for uncontrolled hypertension include:

- The concept of ‘always-on’ effect is certainly an attractive proposal to consider RDN as it could circumvent many of the afore-mentioned impediments to good BP control [60–63]. The TTR (Time in Target Range) for BP achieved is likely to be higher and this metric has been independently associated with decreased major adverse cardiovascular events (MACE) rates (independent of the mean SBP) [64].
  Multiplying the consistent BP-lowering effect with durability of RDN effects of up to 3 years in the Global Symplicity Registry [14], the potential long-term benefits is considerable especially if longer durability could be demonstrated in the future.
- Impact of medication burden
  The majority of patients with resistant or uncontrolled hypertension would be on multiple anti-hypertensive medications. Whilst the likelihood of ‘cure’ with total abolition of need for continued medication is rare, if not impossible, there is a potential reduction of pill burden following RDN.
  Analysis of the SPYRAL-HTN ON-MED pilot trial data using the novel ‘win-ratio method’ clearly showed a reduction in the prescribed medication burden from baseline to follow-up at 6 months [65]. The RADIANCE-HTN TRIO Trial similarly showed sustained BP control at 6 months with reduced anti-hypertensive medications [54]. The reduction in pill burden is likely to influence improved adherence to remaining medications and hence, BP control.

### RDN procedure

**Pre-procedure assessment**

- Pre-procedure investigations

- Renal function test
- Full blood count
- Coagulation profile (optional)
- Crossmatch blood group (optional)
- Renal ultrasound and doppler studies (recommended)
- CTA renal artery (optional)
- MRA renal artery (optional)

**Pre-procedure planning**

- Obtain informed consent (10% of patients may not respond to this therapy. It is not a replacement for BP medications. The aim is to achieve better BP control and reduce cardiovascular risks. It might have the potential to reduce the number of BP drugs.)
- Discontinue agents that could affect renal function (NSAIDS, metformin) for at least 48 hours before the procedure.
- Special care on the doses of prescribed diuretics
- Appropriate hydration prior to the procedure
- Dual anti-platelet therapy (DAPT) interruption not required
- Withhold anticoagulation 48-72 h prior to the procedure
- Continue anti-hypertensive medications as usual or withhold some at physician’s discretion

### The procedure

Percutaneous renal sympathetic denervation is not technically challenging. However, under specific clinical circumstances (e.g., renal insufficiency) or when faced with more complex abdominal aortic anatomy (e.g., tortuosity) some procedural tips may be useful. For further information regarding the procedure, refer to supplementary Appendix 1.

## Perspectives of Asia

Prevalence of hypertension is higher in most of the Asian countries compared to the west. [12, 66–70]. Furthermore, with the rapid rise in population ageing in Asia, Asia will be the oldest region in the world by 2050 [71–73]. Consequently, because prevalence of hypertension increases with age [74, 75], the prevalence of hypertension will increase substantially in Asia and Asia will house the largest number of hypertensives in the world by 2050 [76].

However, the bigger problem is not only the higher prevalence but also the poor control rates in most of the Asian countries compared to the west. [12, 66, 67] Coupled together with several Asian characteristics like higher salt intake, higher salt sensitivity, higher prevalence of early morning BP in Asians than in westerners and the greater impact of BP on CV disease in Asians compared to westerners, greater effort is needed to improve control rates in order to reduce CV mortality and morbidity [12, 77–80]. While anti-hypertensive medications are very effective and safe in lowering BP, many patients are non-adherent to their medications [20, 81–84]. Hence an alternative to getting better BP control needs to be explored and RDN is potentially an effective and safe option for the Asian population [15]

### Future perspectives

With the availability of more recent data since the publication of the last round of hypertension guidelines worldwide, a paradigm shift is expected in the coming guidelines as regards to RDN. The latest WHO guidelines released last year however did not make any mention of RDN except for stating that patients not controlled on 3 drugs (renin–angiotensin–aldosterone system (RAAS) inhibitors, calcium channel blockers (CCB) and diuretics) should be referred to a specialist. However, what is expected is that the recommendation for RDN will be upgraded from the previous Class II recommendation Grade B level of Evidence to Class 1 recommendation with Grade A Level of evidence (for hypertensives who prefer to opt out of medical treatment, for hypertensives who do not favour long-term drug treatment or patients with true drug resistant hypertension). As for patients who are young or obese, with or without, obstructive sleep apnoea, and those with high sympathetic overdrive, a Class II recommendation with Grade C Level of Evidence will be appropriate for the time being until more definitive data from appropriately designed RCTs are available.

### Executive summary

- Hypertension is a prevalent, largely asymptomatic, and potentially dangerous disease (*which may be inadequately controlled in some groups)*. This needs to be recognized and appropriately addressed.
- **Poor control of blood pressure may be due to several factors**:
  - Pseudo-resistant hypertension (includes improper technique, white coat hypertension (WCH), undertreatment, poor medication adherence and clinical inertia) [8, 20, 25–31, 33]
  - Resistant hypertension [2–4, 85, 86]
  - Secondary causes of hypertension. Patients with secondary causes of hypertension might benefit from specific therapy to treat underlying causes rather than RDN
  - Hyperactive renal-sympathetic system (neurogenic hypertension, e.g., obesity, OSA) [87–89]

- This consensus statement takes into consideration the current available data on RDN as a adjunctive treatment modality in poorly controlled/resistant (arterial) hypertension (this includes randomized control trials and global registries [14, 44, 45, 51, 90, 91]
- Poor 24-h control of hypertension is increasingly recognized as a contributor to hypertension complication. RDN can sustainably lower blood pressure over 24-h period according to the SPYRAL-HTN-OFF-MED trial [44].
- The reduction of blood pressure as recorded by ABPM and office systolic blood pressure reading will reduce cardiovascular outcomes [55–58], less hospitalization and bed-occupancy days in Malaysia [59].

RDN can be considered for the following patients. Particular emphasis is made with regard to patient selection which is a key determinant of RDN suitability and success

- **Treatment resistant hypertension**. Treatment resistant hypertension is able to achieve long-term reduction in BP with good safety [90, 92–94].
- **Non-adherence to multiple medications**. *Persistent BP-lowering effect of RDN would thus theoretically reduce the negative consequences of partial and even full non-adherence on clinical outcomes in hypertensive patients* [20, 30, 82].
- **Patient on polypharmacy for multiple comorbidities** [30]
- **Hypertensive patients with hyperactive renal-sympathetic component** [87, 88]

- RDN could serve as a BP-lowering strategy alone or in combination with pharmacotherapy for patients with suboptimal uncontrolled blood pressure [93, 94].
- It is a safe and simple procedure performed under local anaesthesia and sedation

- Renal denervation techniques will continue to evolve using varies type of energy which can further improve renal denervation effectiveness.

- Hypertensive medication will need to be continued following RDN as the blood pressure lowering effect may gradually occur over a period of time (3–6 months and beyond)

**The possible indicators of successful blood pressure lowering after RDN:**

- Patients with baseline office heart rate ≥70 per min, not on anti-hypertensive medications showed greater reduction in mean office, 24-hour, daytime, and night-time SBP for RDN at 3 months [95].
- Younger patients with younger vascular age and low abdominal aortic calcification burden[96], as reported in trials [97].
- Uncomplicated hypertension.

The ongoing SPYRAL ON-MED trial expected to be announced in 2022 is hoped, will provide more data on the promising role of RDN in lowering blood pressure for a selected group of patients continued on concomitant blood pressure medications.

**Managing patient expectations**

1. Hypertensive medications will most likely be continued at current levels for a few months
2. Blood pressure medications usually cannot be stopped and most probably not be reduced. In some cases, these can be reduced. However, blood pressure levels will be better controlled in the longer term.
3. Reduction of blood pressure after post renal denervation will be gradual over a few months to a year
4. Benefits of renal denervation are principally for the reduction of end-organ damage and cardiovascular risk reduction due to better blood pressure control

## Conclusion

- In conclusion, this consensus recommends that for better blood pressure control and reduction of CV risk:

- Successful denervation can be an ***effective adjunctive*** treatment for sustained lowering of blood pressure in hypertensive patients who fulfil the criteria for RDN
- Renal denervation should be considered ***early*** in the management of hypertension
- Should be ***offered*** to patient during consultation in selected group of individuals
- ***Reassurance*** of the RDN procedure should be emphasized
- Patient ***expectations*** should be addressed when discussing RDN
- Informed ***patient preference*** in shared decision making and should be considered for renal denervation consideration.

**Patient preference may be considered in the following:**

1. Young patients who prefer to be on minimal medication
2. Patients who are on multiple medications resulting in high tablet count
3. Patients well motivated to have good and sustained blood pressure control

- It is hoped that this consensus will enable health care providers to understand the science and rationale behind RDN to enable them to identify patients for consideration of this treatment.
- RDN should not be considered as a therapy of last resort but as an initial treatment option.
- Lifestyle modification and pharmacological intervention should remain the mainstay of hypertension management.
- Patient medication adherence should be continually assessed and adjusted as necessary even after successful RDN

## Supplementary information

Supplementary Appendix 1
