# Supplementary material for: 2022 Malaysian Working Group Consensus Statement on Renal Denervation for management of arterial hypertension
Source: Hypertens Res. 2022 Jun 1;45(7):1111–22. doi: 10.1038/s41440-022-00937-w (PMC9192347; doi:10.1038/s41440-022-00937-w)
Supplement: Supplementary file 1 — Supplementary Appendix 1 [file 41440_2022_937_MOESM1_ESM.docx]

# Supplementary Appendix 1:

# 2022 Malaysian Working Group Consensus Statement on Renal Denervation for management of arterial hypertension

## **Appendix 1: How To Do Renal Denervation in Malaysia**

## **AORTOGRAPHY**

- There are a number of techniques for abdominal aortic angiography. One option is to advance a 5F/6F pigtail into the abdominal aorta over a 0.035-inch guidewire superimposing the tip on the transition between the twelfth thoracic and first lumbar vertebrae followed by contrast injection (10-20 cc if digital subtraction angiography, 15-30 cc if conventional angiography).
- Alternatively, a 6 F guide catheter may be advanced over a 0.035-inch guidewire with the guide catheter tip between the 1^st^ and 2^nd^ lumbar vertebrae with injection through the guide catheter while the wire remains in the distal thoracic aorta (this requires the use of a Y-connector prior to angiography). The same guide catheter may then be used to perform selective angiography.
- Important information to obtain: size, take-off, course, tortuosity, bifurcation pattern of the renal arteries, the presence and size of accessory renal arteries and any significant atherosclerosis.


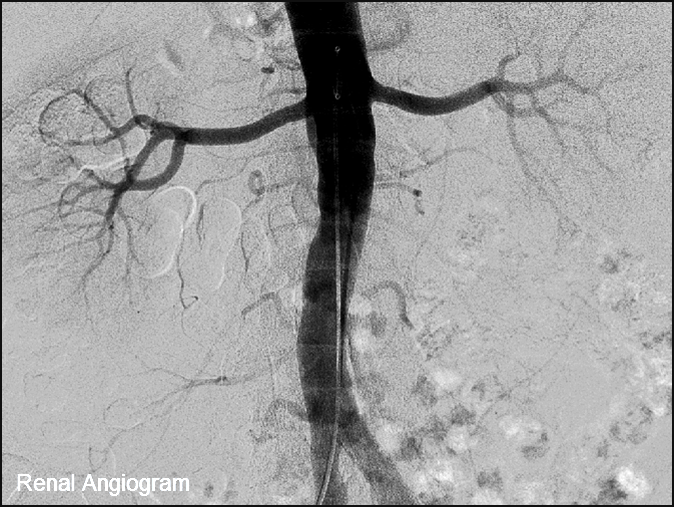


### **SELECTIVE GUIDE CATHETER ENGAGEMENT**

- Once the anatomy is characterised, an appropriate guide catheter is chosen. Most renal arteries have a slightly inferior take-off, thus in most cases, the shape of an internal mammary catheter is suitable for selective engagement.
- In cases of a horizontal take-off, a renal double curve guide catheter or a Judkins right may be suitable.
- In the unusual case of a superior take-off, a multipurpose catheter may provide optimal coaxial alignment.
- In the presence of significant abdominal aortic atherosclerosis, the “no touch” technique is recommended. This entails maintenance of the 0.035-inch guidewire in the distal thoracic aorta while directing the guide catheter tip as close to the renal artery ostium as possible with injections of small amounts of contrast to confirm the close position of the guide catheter tip to the renal artery ostium. Once the catheter tip is near the ostium, the 0.035-inch guidewire is slowly withdrawn allowing the catheter to engage the renal artery more or less passively. This technique avoids atherosclerotic material embolization and minimises the risk of guide catheter-induced renal artery dissection.

### **Adverse anatomy**

- In the presence of iliac and/or abdominal aortic tortuosity, advancement of the renal denervation catheter into the renal artery may be difficult. In this case a long (e.g., 45 cm) sheath may be used to straighten the iliac/aortic vasculature.
- Alternatively, brachial access may be considered.
- If the renal artery has an acute angle inferior take-off, guide catheter engagement and support may be suboptimal. In this case, insertion of a 0.014 or 0.018-inch buddy wire may be helpful to offer more support and a stable guide catheter position. It is important to remove the buddy wire during ablation.

### **Anticoagulation, sedation, analgesia and other medications**

- Heparin 70 units/kg (ACT>250sec)
- Most renal denervation procedures are accompanied by visceral (abdominal, lower back or pelvic) pain. Hence, routine administration of benzodiazepines and opioid analgesics is necessary.
- Typically, intravenous midazolam and fentanyl are used (incremental administration of up to or 4 mg and 150 mcg, respectively, for pain control).
- Sometimes, the use of morphine is necessary. Antiemetics (e.g. 10 mg of intravenous metoclopramide) may be used to prevent opioid-associated nausea.
- Intravenous atropine (1 mg) should be available in case of a vagal reaction as well as flumazenil and naloxone to reverse benzodiazepine and opioid effects in case of respiratory depression

### **The choice of RDN catheter**

- Symplicity Spyral^TM^ catheter


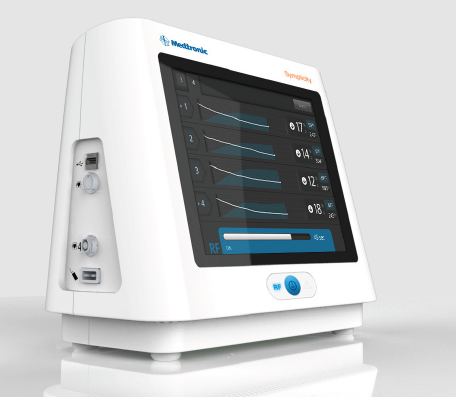

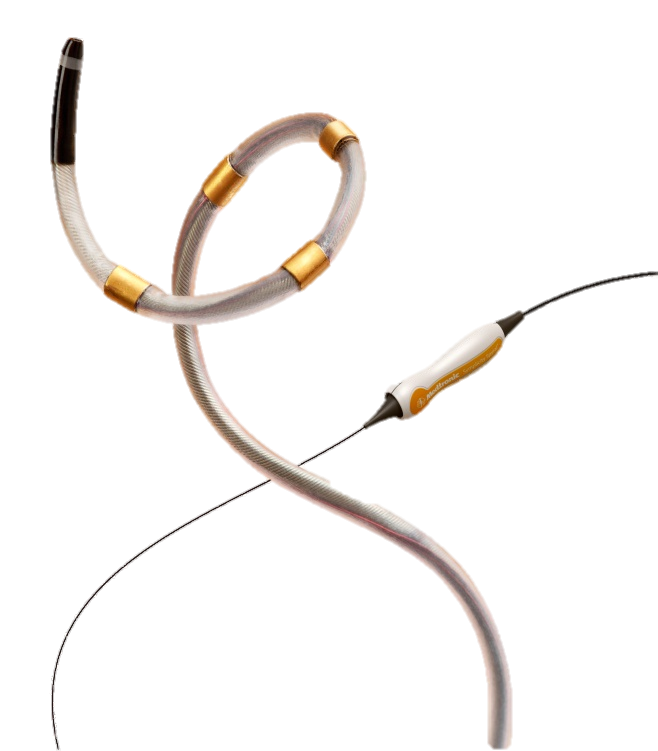


A single catheter that can conform to any vessel size ranging from 3 – 8 mm in diameter.

| **Vessel Diameter (mm)** | **Treatment Length* (mm)** |
| --- | --- |
| 3 | 21 |
| 4 | 20 |
| 5 | 20 |
| 6 | 19 |
| 7 | 18 |
| 8 | 17 |
| * Treatment length: Distance between electrodes 1 and 4 as a function of deployed diameter | |

Symplicity Spyral^TM^ catheter

- Treat renal artery diameters 3 – 8 mm
- Treat main renal arteries, branches, and accessory arteries
- Consistent four-quadrant ablation pattern
- 4F catheter profile
- 6F guide catheter compatible
- 0.014” over-the-wire rapid exchange delivery system
- 60-second simultaneous energy delivery
- Multi-sensor feedback to control energy delivery

Previously, treatment was focused solely on the main body of renal artery. However, studies demonstrated a significantly greater and more consistent treatment effect when both main renal artery and its branches were ablated as compared to main renal artery only.


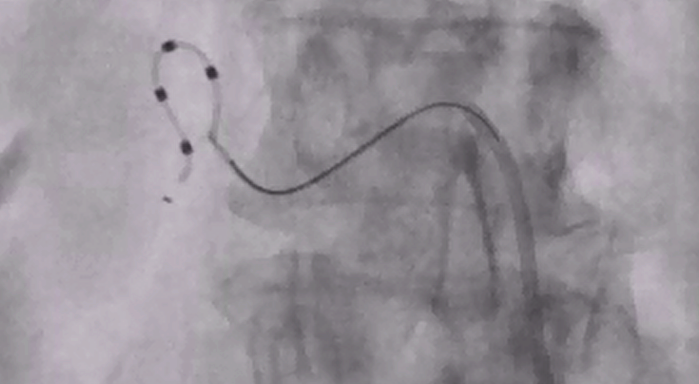

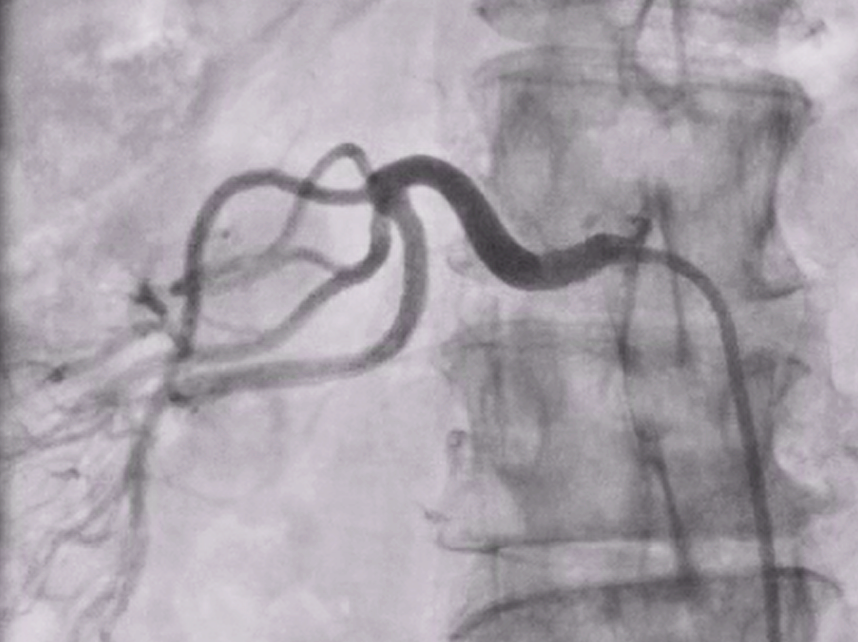


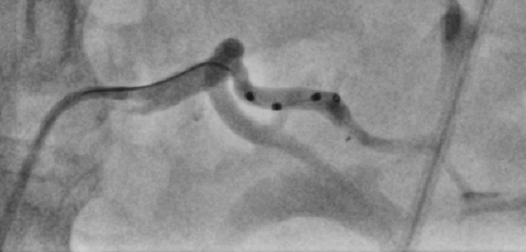

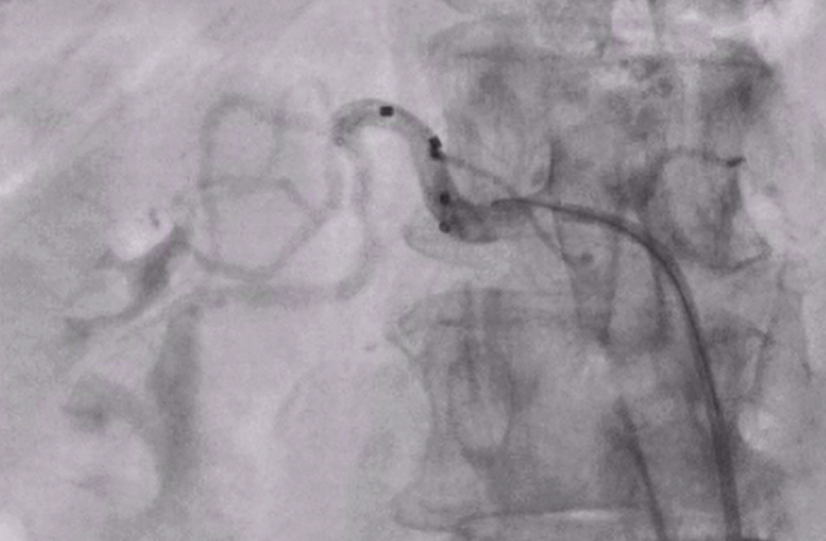


- Impedance may be used to confirm stable wall contact
- Higher impedance may indicate better wall contact
- Stable impedance over a respiratory cycle (Δ < 15-20 ohms) may indicate consistent wall contact
- An impedance drop of >10% is expected and desirable.

### **Paradise catheter**

This consists of a through lumen shaft that delivers ultrasound energy via a piezoelectric ceramic transducer located at the distal end of the balloon catheter which uses sterile circulating water to protect the renal artery approximately 1 mm around the balloon

In a previous swine preclinical study, the Paradise catheter demonstrated nearly 360-degree circumferential ablation, while preventing renal artery medial damage and the maximum and minimum depth of ablation was 11.9 and 1.2 mm.

In the RADIANCE HTN SOLO trial, the duration of the ablation was 7 seconds and the mean number of ablations was 5.4±1.0 and they were performed at a distance 5 mm apart with an average total time of treatment 37.9±6.7 seconds, and total procedure time of 72 minutes.

### **Other useful tips and trick**

- Carbon dioxide angiography in the setting of advanced renal insufficiency or prior contrast allergy
- Prior to renal denervation catheter delivery, some operators routinely administer a vasodilator intra-arterially (e.g., 200-400 micrograms of intra-arterial nitroglycerine, 200 mcg of nicardipine, 100 mcg of verapamil or 100 mcg of nitroprusside) in an attempt to prevent or minimise vasospasm.
- When a renal artery stenosis is encountered the renal artery stenosis may be treated with balloon angioplasty first and renal denervation performed at a later time.
- It is important to avoid ablation in a stented segment as this may cause significant heat-related tissue injury.
- Equipment that allows renal artery stenting should be readily available in the rare scenario of guide or renal denervation catheter-induced renal artery dissection.
- The use of digital subtraction angiography may allow a smaller amount of contrast.
- Pulse oxymetry for continuous oxygen monitoring as well as reversal agents (1 mg of intravenous flumazenil and 2 mg of intravenous naloxone) and emergency airway management equipment (including for intubation) in case of excessive sedation with respiratory compromise must be on standby.

## **Post procedure follow-up**

Groin management is similar to coronary angioplasty

Patient is kept overnight

No DAPT is given at discharge

Usually no adjustment of BP medication at discharge (Physician’s discretion)

1-3 monthly follow ups for the first 6 months

Subsequently 4-6 monthly

Renal profile at first month then every 4-6 months

Abdominal ultrasound at physician’s discretion
